# Machine Learning-Guided Prediction of Central Anterior Chamber Depth Using Slit Lamp Images from a Portable Smartphone Device

**DOI:** 10.3390/bios11060182

**Published:** 2021-06-05

**Authors:** David Chen, Yvonne Ho, Yuki Sasa, Jieying Lee, Ching Chiuan Yen, Clement Tan

**Affiliations:** 1 Department of Ophthalmology, National University Hospital, Singapore 119228, Singapore; clement_wt_tan@nuhs.edu.sg; 2 Keio-NUS CUTE Center, Smart Systems Institute, National University of Singapore, Singapore 117602, Singapore; yvonne.ho@nus.edu.sg (Y.H.); yukisasa@keio.jp (Y.S.); idmleej@nus.edu.sg (J.L.); didyc@nus.edu.sg (C.C.Y.); 3 Division of Industrial Design, National University of Singapore, Singapore 117356, Singapore; 4 Yong Loo Lin School of Medicine, National University of Singapore, Singapore 117599, Singapore

**Keywords:** narrow angle, screening, portable, machine learning, smartphone

## Abstract

There is currently no objective portable screening modality for narrow angles in the community. In this prospective, single-centre image validation study, we used machine learning on slit lamp images taken with a portable smartphone device (MIDAS) to predict the central anterior chamber depth (ACD) of phakic patients with undilated pupils. Patients 60 years or older with no history of laser or intraocular surgery were recruited. Slit lamp images were taken with MIDAS, followed by anterior segment optical coherence tomography (ASOCT; Casia SS-1000, Tomey, Nagoya, Japan). After manual annotation of the anatomical landmarks of the slit lamp photos, machine learning was applied after image processing and feature extraction to predict the ACD. These values were then compared with those acquired from the ASOCT. Sixty-six eyes (right = 39, 59.1%) were included for analysis. The predicted ACD values formed a strong positive correlation with the measured ACD values from ASOCT (R^2^ = 0.91 for training data and R^2^ = 0.73 for test data). This study suggests the possibility of estimating central ACD using slit lamp images taken from portable devices.

## 1. Introduction

Angle closure glaucoma is a cause of major visual impairment in Asia; 87% of patients with angle closure glaucoma globally are Asian [1]. The prevalence of patients with all occludable angles (including primary angle closure and primary angle closure glaucoma) is estimated to be 6.3% in Singaporeans aged 40 years old and above [2], of whom 79% may not have been diagnosed before [3]. Age and sex standardized incidence of acute angle closure glaucoma (AACG) was reported as being up to 15.5 cases/100,000 person-years among Chinese Singaporeans [4,5]. In addition, only 25–35% of AACG in Asian people cause symptoms [3,6,7]. The direct cost of treatment for AACG in Singapore has been estimated to be between US$879.45 to US$2576.39 over five years [8].

The advent of anterior segment optical coherence tomography imaging (ASOCT) has made anterior chamber assessment both quantitative and objective. There has been a rapid development and maturation of OCT imaging technology over the past two decades, and it has seen a significant increase in clinical applications in anterior segment conditions, from cornea to dry eyes to glaucoma assessment [9]. Common parameters used to describe the features of the anterior chamber angle include the angle opening distance (AOD), the anterior chamber area (ACA), and the trabecular-iris space area (TISA), with AOD750 being the best performer in a community-based screening study [10]. However, ASOCT machines are expensive and bulky, and may only be available in specialist eye clinics.

**Figure 1:**
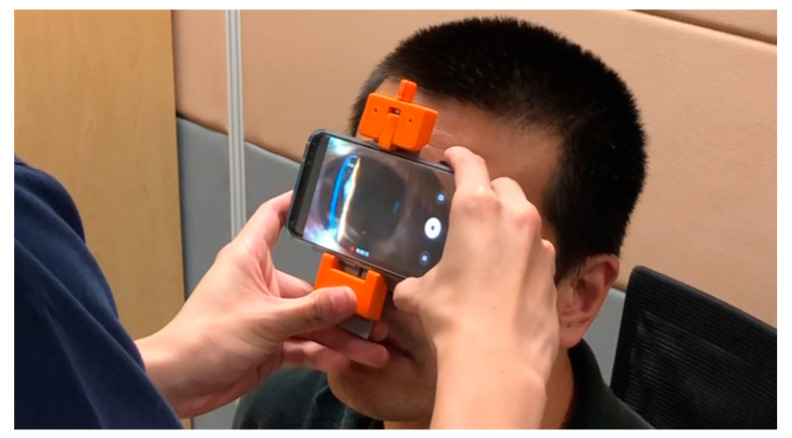
Sample of a MIDAS device in use.

Artificial intelligence (AI) has seen tremendous breakthroughs in ophthalmic imaging in recent years [11], especially with respect to fundal imaging for the diagnosis of diabetic retinopathy [12,13], age-related macular degeneration [14,15], and glaucoma [16]. Machine learning techniques have also been employed more recently to ASOCT to automatically detect patients at risk of gonioscopic angle closure [17,18,19]. This provides the opportunity to automatically and conveniently screen patients at risk of angle closure disease in high-risk populations in lieu of the labour-intensive gonioscopy. However, there are limited options available for standardized angle screening using portable devices. In this study, we describe our exploration using machine learning to predict anterior chamber depth (ACD) through slit lamp images taken with a portable smartphone slit lamp device (MIDAS; Figure 1).

## 2. Materials and Methods

### 2.1. Patient Recruitment and Image Capture

Here, 70 eyes of 70 patients were recruited from June 2018 to February 2019. After excluding two eyes because of poor image quality and two because of inadequate ASOCT images, a total of 66 eyes (right = 39, 59.1%) were included.

This was a prospective, single centre clinic-based, digital imaging validation study. Prospective patients in the outpatient eye clinic were identified by a research assistant prior to their visit, and were recruited after informed consent at the end of their consultation. The inclusion criteria of these patients were as follows:

- Willing and able to participate in study
- Be at least 60 years old (inclusive)
- Had not had prior intraocular surgery or laser procedures to the eye
- Be fit enough for keep eyes open for adequate image acquisition
- Not have concurrent eye pathologies that may obscure photo-taking of the eye
- Not have previous laser or surgical glaucoma interventions

The research followed the tenets of the Declaration of Helsinki and was approved by the institutional domain specific review board (DSRB). Informed consent was obtained from all subjects after an explanation of the purpose and possible consequences of the study.

### 2.2. Image Capture Protocol

After acquiring informed consent, patients were brought to another room for sequential image capture with the following set-up, as described below.

- Set-up A: Smartphone (Samsung^®^ Galaxy S7, Seoul, South Korea) with a MIDAS portable slit lamp mount prototype.
- Set-up B: Corneal anterior segment non-invasive three-dimensional swept source imaging system (Tomey^®^ SS-1000 CASIA ASOCT, Nagoya, Japan).

Set-up A was performed in a dimmed room to simulate mesopic conditions similar, but not identical, to those used for van Herick grading [20]. The MIDAS device is a lightweight portable slit lamp prototype that utilizes a smartphone camera to capture anterior segment images. It is a non-contact device that comprises a light-emitting diode (LED) module fitted behind an optical slit and achromatic condensing lenses to produce an incident light of 45°, with the beam measuring 1 mm wide by 15 mm tall. It contains a clamp that could be fitted to most regular sized smartphones and is powered by the smartphone’s battery (Figure 1). When assembled with a compatible smartphone, the set-up provides 10× image magnification through a macro lens and focuses at a distance of 21 mm. The MIDAS device is currently not commercially available.

**Figure 2:**
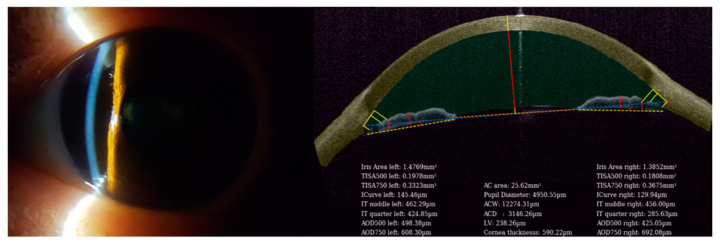
A sample image obtained from the MIDAS instrument and its corresponding anterior segment optical coherence tomography image.

A standardized image capture protocol is performed using Set-up A, including image capture in the same room under identical mesopic conditions. With the patient looking forward, the device is advanced squarely towards the eye of interest, parallel to the frontal plane of patient’s eye. The incident light beam is then focused over the anterior iris surface in the mid-peripheral iris (Figure 2, left). Care is taken to ensure the light beam is not too central (where it would be interrupted by the pupil) or too peripheral (where it might be obscured by the peripheral arcus). Once focused, an anterior segment slit lamp image is captured using the smartphone camera app, and the resultant file is saved as a camera Raw image on the smartphone (Figure 2, left). Depending on patient cooperation, multiple images may be captured in approximately 1 mm steps from nasal to temporal, and the best image would be selected by the performing technician. For the purposes of this study, the corneal curvature was assumed to be prolate ellipsoid; no formal keratometry or corneal topography was performed.

Following image capture of the undilated eye(s), patients were moved to the adjacent room for ASOCT capture using Set-up B. If both eyes were eligible, non-mydriatic images were captured from both eyes, but only one eye was selected for analysis – by default, the eye with an image of a better quality was selected. Through this, two sets of images of the same eye were captured—anterior segment slit lamp images from set-up A (Figure 2, left) and ASOCT images from set-up B (Figure 2, right).

Sixteen equally-spaced angle images (eight meridians) per eye were manually marked by a grader to identify the scleral spur for the automatic calculation of the relevant anterior chamber parameters [21]. Separately, de-identified ASOCT images from Set-up B were annotated manually using CASIA software by a single grader. The relevant anterior chamber parameters were extracted from ASOCT and can be used as the reference for training and testing the machine learning model. These include, but are not limited to, the following: angle opening distance at 500 µm (AOD500), trabecular-iris space area at 500 µm (TISA500), angle recess area (ARA), and central anterior chamber depth (ACD).

### 2.3. Image Feature Extraction and Application of Machine Learning Techniques

The Van Herick method is a technique to screen for angle closure based on the width of the corneal slit image and slit image on the iris generated by the slit lamp. Using the same principles behind the Van Herick method, the width between the corneal and iris slit in the slit lamp images obtained from the smartphone (Figure 2, left) can also be used to relate to the angle closure and ACD. These dimensions from the slit lamp image captured by the smartphone and the corresponding ACD obtained from the patient’s ASOCT image (Figure 2, right) were used to train the machine-learning model.

The images captured on the smartphone camera were processed by independent operators with no prior knowledge of patients’ medical condition. Adobe Illustrator (version CC 2019) was used to extract the dimensions related to ACD by measuring the distance, in pixels, between various landmarks of the image. The landmarks were kept consistent for all of the eye images and were annotated for future reference.

The various dimensions extracted from the smartphone camera image and the corresponding ACD values from the ASOCT images were then used to train a Random Forest Regression model to predict ACD. Random Forest [22] uses multiple decision trees generated using bootstrapping, where a randomly selected subset of data is used to train an individual decision tree. The outputs of the individual decision trees were then aggregated to get the output of the model.

The Random Forest model was trained using the entire sample set. However, the estimate of the error was evaluated using out-of-bag data. Out-of-bag data for a tree refers to data that were not used to generate that particular decision tree. The out-of-bag data for each tree was used as test data, and these predicted values were aggregated for every sample data point when it was an out-of-bag sample.

For our Random Forest regression model, the number of trees used was 11, with a minimum leaf sample of 1. For each decision tree, the data were randomly split into 0.6 of the samples used as the training subset and 0.4 of the samples as out-of-bag data, which were then used to estimate the error.

For regression, there are several loss functions that can be employed, such as mean square error, mean absolute error, and structural similarity index [23,24]. The loss function, or error criterion, used in training was mean absolute error (MAE), which is given by Equation (1),

(1) $$MAE=\frac{\sum_{i=1}^{n}\left|y_{i}-{\hat{y}}_{i}\right|}{n}\;$$

where *n* is the total number of samples, $y_{i}$ the observed data, and ${\hat{y}}_{i}$ the predicted data.

These hyperparameters and loss functions were chosen to minimize out-of-bag error scores (out-of-bag error score = 1–coefficient of determination) which reflects the model ability to generalize. The Random Forest model was implemented using Scikit-learn (ver 0.23.2).

## 3. Results

**Figure 3:**
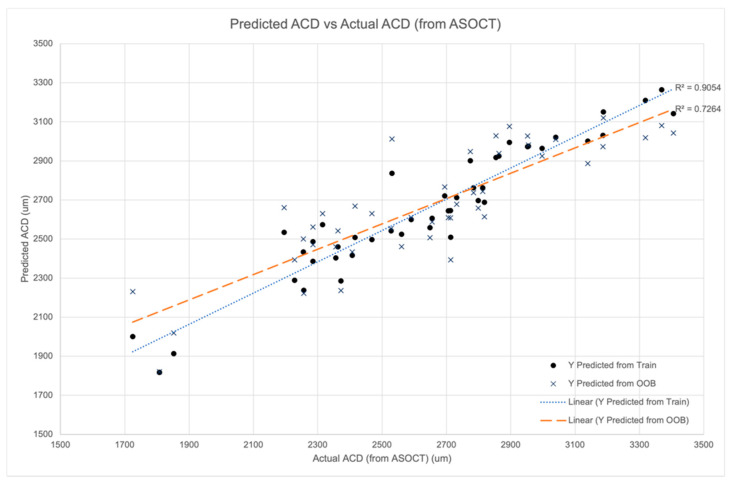
Training data validation. Central anterior chamber depth (ACD) values predicted from the training data vs. actual ACD from ASOCT.

Figure 3 shows the relationship between the Random Forest model predicted central ACD values and their corresponding ASOCT-measured central ACD. The values predicted by the training data inputs had a positive linear correlation with a coefficient of correlation R^2^ of 0.91 which gave the training error. The out-of-bag sample data R^2^ was 0.73. Tuning the model hyperparameters to increase the training data R^2^ would result in higher out-of-bag data prediction errors due to overfitting.

The root mean square error (RMSE), given by Equation (2), is used to compare the training set and out-of-bag set.

(2) $$RMSE=\sqrt{\frac{\sum_{i=1}^{n}{\left(y_{i}-{\hat{y}}_{i}\right)}^{2}}{n}}$$

where *n* is the total number of samples, $y_{i}$ the observed data, and ${\hat{y}}_{i}$ the predicted data. The RMSE of the training and out-of-bag data are given in Table 1.

**Figure 4:**
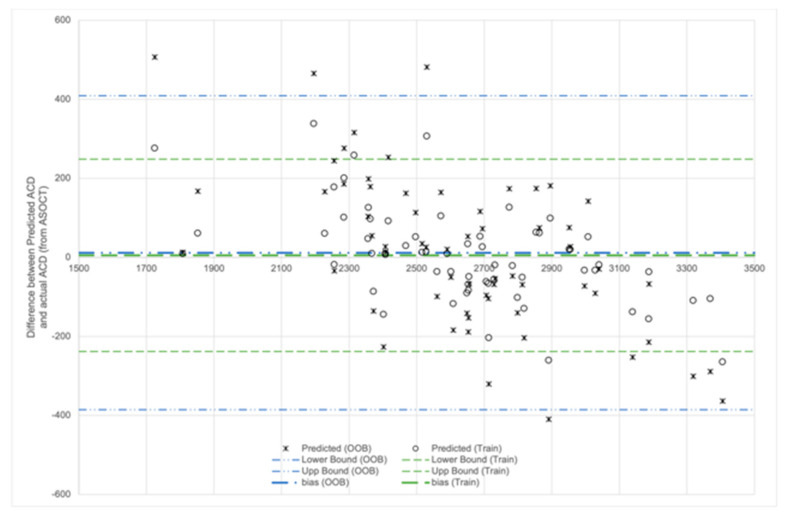
Bland–Altman plot showing ASOCT-derived ACD and predicted values from the training inputs and the out-of-bag samples.

Figure 4 shows the Bland–Altman plot of the predicted ACD values from the training data and out-of-bag data, against ACD obtained from ASOCT. Most of the predicted values were within 200 µm of the actual ACD from ASOCT. The model tended to overestimate the samples with smaller ACD, and underestimate the value of the samples of samples with larger ACDs.

The features importance, which is calculated based on Gini-impurity, are given in Table 2. The input features are derived from dimensions measured between various landmarks of the eye image, which was captured on a smartphone with a portable slit lamp.

## 4. Discussion

This pilot study demonstrated that using images captured from a portable slit lamp device, it was possible to use machine learning to predict the central ACD of our patients. To our knowledge, this is the first study of its kind using machine learning on a mobile slit lamp device.

Angle closure glaucoma is a cause of major irreversible visual loss in Asia [1]. However, patients with this condition are typically asymptomatic, making the identification of this condition difficult in the community. The diagnosis of angle closure disease usually requires clinical examination with a slit lamp, with gonioscopy and additional anterior segment imaging including ASOCT. Subjective clinical assessment such as van Herick grading has only moderate repeatability in trained graders [25,26]. In addition, there is currently no objective screening modality in the community—healthcare practitioners could use a pen torch or handheld slit lamp, but these assessment methods are subjective and are also dependent on clinical experience. Machine learning has previously been applied to ASOCT to automatically measure anterior chamber features with variable success [17,18,19]. However, ASOCT machines are bulky and expensive, making them unsuitable for deployment in a community-based setting. The van Herick method has been proposed as a simple triage test before gonioscopy [27], but it has been reported to have varying sensitivities and specificities, with the lowest being 53% [28] and 57% [6], respectively. In addition, the van Herick method is technically harder to perform in a mobile setting using a portable slit lamp. Screening with a pen torch would be expected to have even poorer sensitivities and specificities.

Our novel portable slit lamp could be used with most smartphones of regular dimensions, and with this pilot study, there is promise of an objective AI-driven diagnostic capability for angle screening, for the first time made for a portable device. Patients who are identified to be at risk of angle closure disease with this technology could then be referred to a tertiary eye centre for further evaluation of possible angle closure. Further studies would be required to refine the AI algorithm to predict other relevant anterior chamber parameters and to define thresholds for making recommendations for further formal angle closure assessment.

In this exploratory study, we were limited by the small sample size (*n* = 66). The out-of-bag sample prediction R^2^ was 0.73 with a bias of 955.18, which may be due to the small sample size. Manual marking of image landmarks by human graders could also introduce inaccuracies. To improve the model, a larger sample size is needed, with a better-balanced distribution of central ACD values, and the Random Forest hyperparameters need to be tuned again. Other machine learning methods for regression, such as neural networks, can also be studied and evaluated with the aim of improving prediction outcomes. Automatic feature extraction using image processing methods could be employed to reduce inter- and intra-operator errors in the manual extraction of features from images obtained by smartphones.

The prediction is currently limited to central ACD, but the prediction of other anterior chamber parameters (AOD500, TISA500, ACA, etc.) would be expected in subsequent studies of larger sample sizes. In addition, not all patients underwent gonioscopy for this study, as the clinicians attending to the patients were not directly involved in this study and thus there was no standardized comprehensive angle assessment. However, the aim of this study was to predict central ACD in all patients and, in this regard, the classification of narrow angles was of secondary importance. Lastly, this study was performed on a uniformly East Asian population (all patients were Chinese) aged 60 years and above, thus the results may not be extrapolated to other dissimilar populations. More studies would be required to refine the model and compare it with current clinical standards of community-based angle screening.

## 5. Conclusions

In conclusion, we have developed a new method of predicting central ACD using a portable smartphone slit lamp device aided by machine learning.

## Figures and Tables

**Table 1:** Metrics of the training and out-of-bag data set.

	Training Set	Out-of-Bag Samples
Coefficient of Correlation, R^2^	0.91	0.73
Bias	542.85	955.18
RMSE	122.33	200.03

**Table 2:** Features importance (normalized) calculated based on Gini-impurity.

Dimensions	Feature Importance (Normalized)
A	0.15
B	0.13
C	0.18
D	0.16
E	0.16
F	0.10
G	0.11

## Data Availability

The data presented in this study are available on request from the corresponding author. The data are not publicly available due to institutional restrictions.
